# L-arginine impact on inflammatory and cardiac markers in patients undergoing coronary artery bypass graft: a systematic review and meta-analysis of randomized controlled trials

**DOI:** 10.1186/s12872-024-04318-8

**Published:** 2024-11-13

**Authors:** Zahra Mohammadi, Mahdi Ravankhah, Mohammad Ahmadi, Omid Keshavarzian, Isaac Azari, Mozhan Abdollahi, Mehdi Rezaei, Hamed Akbari

**Affiliations:** 1 Student Research Committee, Fasa University of Medical Sciences, Fasa, Iran; 2 Student Research Committee, Shiraz University of Medical Sciences, Shiraz, Iran; 3 Students’ Scientific Research Center, Tehran University of Medical Sciences, Tehran, Iran; 4 Shiraz School for Medicine, Shiraz University of Medical Sciences, Shiraz, Iran; 5 Student Research Committee, Yasuj University of Medical Sciences, Yasuj, Iran; 6 Student Research Committee, School of Medicine, Shiraz University of Medical Sciences, Shiraz, Iran; 7 Department of Cardiology, Fars-Iranian Heart Association, Fars Society of Internal Medicine, Shiraz, Iran; 8 Cardiovascular Research Centre, Department of Pediatrics, Faculty of Medicine and Dentistry, University of Alberta, Edmonton, Alberta Canada

**Keywords:** Arginine, Cardioplegia, Ischemia-reperfusion injury, Coronary artery bypass surgery, Inflammation

## Abstract

**Background:**

Numerous studies have explored the effects of L-arginine, whether administered in the form of a supplement or through infusion during cardioplegia, on cardiac and inflammatory markers in individuals undergoing coronary artery bypass grafting (CABG). However, these studies presented contradictory findings. Consequently, the objective of this study was to investigate the impact of l-arginine on these markers by analyzing available randomized controlled trials (RCTs).

**Methods:**

We performed an extensive search across various databases, including Embase, Medline/PubMed, Web of Science, Scopus, Cochrane Library, and Google Scholar, covering research published until December 2023. To analyze the mean changes in inflammatory and cardiac markers between the L-arginine and control groups, we calculated the weighted mean difference (WMD) along with the corresponding 95% confidence interval (CI) using a random-effects model.

**Results:**

A total of 393 RCTs were identified during the initial search. After screening and selection, 7 trials were included. In a meta-analysis of three trials that reported troponin T levels, we found a significant impact of L-arginine on reducing troponin T levels (WMD = -0.61 ng/ml; 95% CI: -1.07, -0.15). Our analysis also showed that L-arginine had a noticeable impact on decreasing interleukin-6 (IL-6) levels (WMD = -7.72 pg/ml; 95% CI: -15.05, -0.39). However, we found no considerable impact of L-arginine treatment on creatine phosphokinase-MB (CPK-MB), tumor necrosis factor-alpha (TNF-α), and troponin I compared to the placebo groups.

**Conclusions:**

Our findings suggest that L-arginine may benefit patients undergoing CABG, as it helps reduce inflammatory reactions and limits myocardial ischemia. This study registered in the PROSPERO database (Registration No. CRD42024508341).

## Introduction

Cardiac operations, including coronary artery bypass graft (CABG) surgery, have become more common due to the aging population and prevalence of cardiovascular disease [1]. While surgical stress during these procedures can initially benefit healing and recovery, persistent or severe inflammatory reactions can lead to organ failure and even death [1, 2]. During CABG surgery, nitrative and oxidative stress can cause damage to cells, particularly during the process of re-energizing previously damaged mitochondria [3]. Endothelial injury has been identified as a significant underlying factor in postoperative cardiovascular dysfunction, with potential implications for adverse cardiovascular outcomes [4, 5]. While current cardioplegic techniques are effective for low-risk patients, high-risk patients may benefit from innovative additives that can enhance cardioplegic effects, reduce morbidity and mortality, and improve outcomes following CABG surgery [6].

The utilization of L-arginine in cardioplegia has been on the rise as a means to safeguard the heart by facilitating the production of nitric oxide (NO). This results in vasodilation, reduced endothelial dysfunction [7], and enhanced coronary blood flow, all of which collectively contribute to its cardioprotective effects [8]. Moreover, previous investigations have provided evidence that L-arginine has an advantageous influence on both insulin sensitivity and endothelial function, not only in subjects without any health conditions but also in those with type 2 diabetes [9–11]. Some studies suggest that oral administration of L-arginine can decrease the levels of NO and ameliorate endothelial dysfunction in hypertensive individuals [12, 13]. NO plays a crucial role in regulating various cardiovascular mechanisms, including vascular structure, tone, and interactions between cells within blood vessels [10]. Extensive research has emphasized the vasodilatory effects of NO and its ability to preserve endothelial function, underscoring its importance in protecting against ischemia-reperfusion damage [14–16]. However, there are conflicting findings regarding the impacts of L-arginine on inflammatory and cardiac biomarkers. These studies used various doses of L-arginine doses.

To date, the advantageous impact of L-arginine on inflammatory and cardiac indicators have remained uncertain. Thus, in this systematic review and meta-analysis, we explored randomized controlled trials (RCTs) aiming to assess L-arginine impact on myocardial stress, considering myocardial cytokines (interleukin-6 (IL-6) and tumor necrosis factor-alpha (TNF-α)) and myocardial ischemia, indicated by troponin T concentration and creatine phosphokinase-MB (CPK-MB) levels, specifically among patients who underwent CABG surgery.

## Materials and methods

All aspects of the present systematic review and meta-analysis, including data processing, analysis, and reporting, were carried out in accordance with the PRISMA guidelines. We aimed to investigate the link between L-arginine and different inflammatory and cardiac biomarker levels.

### Search strategy

A thorough and extensive search of medical databases, including MEDLINE, PubMed, Embase, Web of Science, Scopus, Cochrane library, and Google Scholar, was conducted to find relevant RCTs reporting on the impact of L-arginine on inflammatory and cardiac markers in subjects undergoing CABG surgery. Our search spanned from the inception of these databases through December 21, 2023. The search strategy employed the following pattern to ensure comprehensive coverage of relevant studies: (key terms for the population) AND (key terms for l-arginine) AND (key terms for outcomes) AND (key terms for study design). The search was performed without any limitations on the language, publication year, study period, or sample size. Our search encompassed these keywords and MeSH terms: (1) “Coronary Artery Bypass” OR “Coronary Artery Bypass Grafting” OR “Bypass Surgery, Coronary Artery” OR “Bypasses, Aortocoronary” OR “Bypass, Aortocoronary” OR “Aortocoronary Bypasses” OR “Aortocoronary Bypass” OR “Bypass, Coronary Artery” OR “Coronary Artery Bypass Surgery” OR “Coronary Artery Bypasses” OR “Bypasses, Coronary Artery” OR “Artery Bypasses, Coronary” OR “Artery Bypass, Coronary”; (2) “Arginine” OR “L-arginine” OR “Arg” OR “L-Arg”; (3) “Interleukin-6” OR “Interleukin-1” OR “C-reactive protein” OR “Inflammatory factors” OR “Tumor necrosis factor-alpha” OR “VCAM” OR “ICAM” OR “high sensitivity C-reactive protein” OR “hs-CRP” OR “tumor necrosis factor-alpha” OR “TNF-alpha” OR “interleukin 8” OR “interleukin2” OR “Creatine Kinase, MB Form” OR “Troponin T” OR “Troponin I” OR “Troponin-I” OR “Troponin I1” OR “Troponin I^2^” OR “Troponin I3” OR “Troponin T3” OR “Troponin T2” OR “Troponin T1” OR “Troponin-T” OR “Myocardial Creatine Kinase” OR “interleukin” OR “Creatine Kinase, Myocardial” OR “Kinase, Myocardial Creatine” OR “Isoenzyme CPK-MB” OR “CPK-MB, Isoenzyme” OR “Isoenzyme CPK MB MB Creatine Kinase” OR “Creatine Kinase, MB” OR “Kinase, MB Creatine” OR “monocyte chemoattractant protein-1, human” OR “MCP1 protein, human”; (4) “Clinical Trials as Topic” OR “intervention” OR “intervention*” OR “trial” OR “randomized” OR “randomized” OR “placebo” OR “random” OR “randomly” OR “assignment” OR “clinical trial” OR “parallel” OR “cross-over” OR “RCT.” To increase the sensitivity of our search, we reviewed the reference lists of all recently published meta-analyses and included RCTs to identify any other related research.

### Eligibility criteria

We included RCTs that (1) specifically investigated the impact of L-arginine on inflammatory and cardiac biomarkers, (2) involved human subjects who underwent CABG surgery, (3) enrolled adults over 18 years of age, (4) administered L-arginine either preoperatively or intraoperatively, and (5) were written in English. Conversely, we excluded investigations if they were (1) animal studies, review articles, editorials, letters to the editor, abstracts of congress presentations, peer-reviewed manuscripts, case series and case reports, in vivo or in vitro, commentaries, or abstracts lacking full text. (2) Were designed as single-arm trials or lacked a control group. (3) Provided insufficient data for analysis. (4) Failure to disclose or present desired outcomes. No restrictions were placed on publication date for study identification. Two independent authors screened the titles, abstracts, and full texts of the eligible papers based on the inclusion criteria. Any disagreements between investigators were resolved through the involvement of a third author.

### Data extraction

Two investigators (MA and OK) independently extracted the data using an Excel spreadsheet for data abstraction. Discrepancies were resolved through consensus using a standardized abstraction checklist to record the data in each study. Any disagreements were resolved through discussion with another senior author (HA) Gathered data encompassed the author’s name, publication year, site (country), population characteristics (including the number of participants, mean age, sex, and sample sizes in the intervention and control groups), intervention duration, intervention type, study design, L-arginine dose, timing of variable measurements, and key outcome data on the mean and standard deviation (SD) of CPK-MB, troponin T, interleukin-6, and TNF-α in both groups. We requested any relevant missing data from the corresponding author via email, with two reminders sent at two-week intervals of two weeks. In certain instances, articles were retrieved multiple times owing to the presence of subset data.

### Quality assessment

**Fig. 1:**
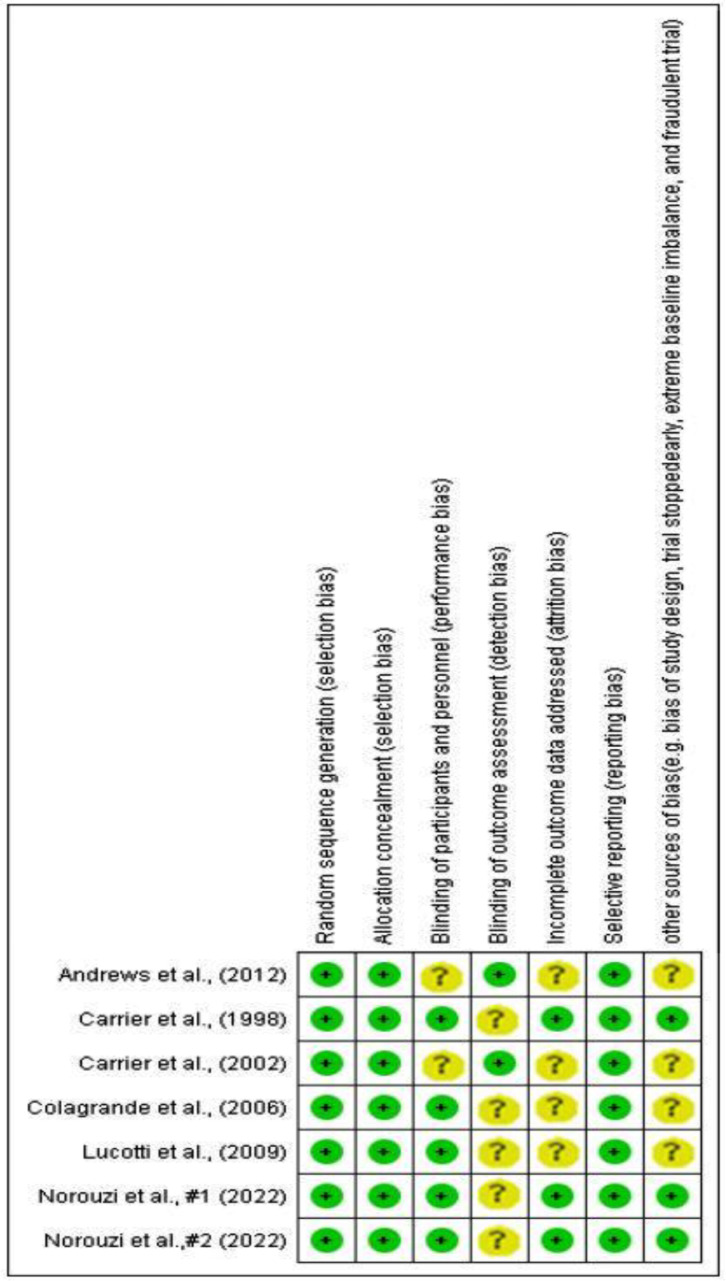
Quality assessment of included studies

The quality of the selected studies was thoroughly evaluated using the Cochrane Risk of Bias (RoB) tool. The assessment encompassed various aspects, such as randomization generation, allocation concealment, blinding of subjects and outcome assessment, incomplete outcome data, selective outcome reporting, and other potential sources of bias. The findings of the quality assessment of the selected studies are shown in Fig. 1. Disagreements between reviewers were resolved through discussion. The assessment for each item involved determining the risk of bias as either ‘low risk,’ ‘high risk,’ or ‘unclear risk.’ The category of ‘unclear risk” indicated a lack of information or uncertainty regarding the potential for bias in that specific aspect of the study. In terms of selection bias (random sequence generation and allocation concealment) and reporting bias (selective reporting), all studies were rated as low risk. In terms of performance bias (blinding of participants and personnel), the majority of studies were classified as low risk, except for those conducted by Andrew et al. [17]. and Carrier et al. [18]. , which were considered unclear risk. Regarding detection bias (blinding of outcome assessment), all studies were labeled as unclear risk, except for those conducted by Andrew et al. [17]. and Carrier et al. [18]. , which were categorized as low risk. Additionally, for attrition bias (incomplete outcome data addressed) and other sources of bias, all studies were classified as unclear risk, except for the studies conducted by Carrier et al. [19]. and Norouzi et al. [1, 20]. , which were rated as low risk. Based on these assessments, three of our studies were considered low-risk [1, 19, 20], while four were deemed high-risk [7, 10, 17, 18].

### Statistical analysis

We extracted the mean values of alterations in inflammatory and cardiac markers, along with their SDs, from the intervention and control groups. These values were used to compute the mean difference (MD) and the corresponding standard errors, serving as pooled effects for the outcome measures. Additionally, a random-effects model using the DerSimonian-Laird method was employed to compute weighted mean differences (WMDs) with 95% confidence intervals (CIs) to assess between-study variation in the meta-analysis. The SD of mean differences was calculated using the formula SD = √[(SDpre-treatment)2 + (SDpost-treatment)2 – (2R × SDpre-treatment × SDpost-treatment)], where R represents the association between the pre- and post-treatment scores. An assumed correlation value of *R* = 0.5 was used to compute the SD. We used Cochran’s Q test and the I-squared (I^2^) index to detect potential source heterogeneity. A p-value < 0.1 for the Q test or I^2^ > 50% indicated significant heterogeneity among the studies. We performed a sensitivity analysis to examine the potential impact of specific individuals or groups of studies on the overall effect size. Begg’s and Egger’s regression asymmetry tests were used to assess publication bias. Statistical significance was set than 0.05. Statistical analyses were performed using STATA software (version 16.0; Stata Corp, College Station, TX, USA).

## Results

### Search results and study selection

**Fig. 2:**
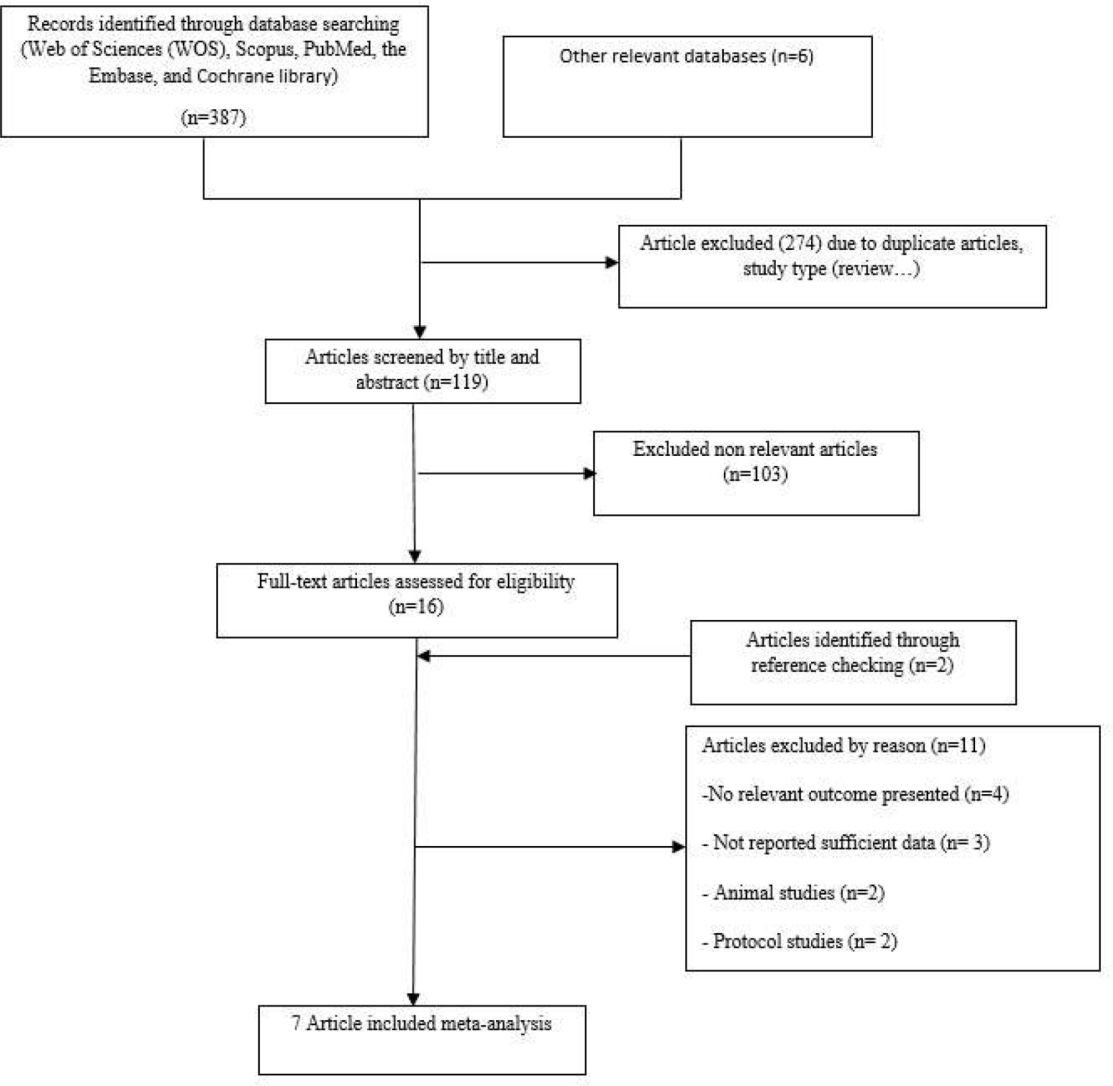
Flowchart of study identification and selection process

Figure 2 presents the PRISMA flowchart, visually outlining the sequential process of conducting a literature search and selecting studies. After a comprehensive search of electronic databases, a total of 393 potentially relevant articles were identified. After omitting articles based on study type and eliminating duplicates, 119 articles remained. Notably, 103 articles were excluded after screening their titles and abstracts. Ultimately, 16 publications remained for further evaluation after reading the full text. During this stage, two additional articles were included based on references from the selected articles. Additionally, 11 articles were excluded after applying the predefined inclusion and exclusion criteria: four articles were deemed irrelevant to the outcomes of interest, three articles lacked sufficient data reporting, two articles were identified as animal studies, and two articles were removed because they were protocol studies. In total, we included seven eligible RCTs in our systematic review and meta-analysis [1, 7, 10, 17–20].

### Study characteristics

Table 1 provides an elaborate account of the attributes and features of the studies included in the analysis. One study [7] specifically investigated the impact of l-arginine on inflammatory and cardiac markers. Additionally, four trials [17–20] assessed cardiac markers, while two trials [1, 10] distinctly evaluated inflammatory markers. RCT publication dates from 1998 to 2022. Two RCTs were performed in Iran [1, 20], while the remaining studies were carried out in Italy [7, 10], Canada [18, 19], and Australia [17]. A total of 508 participants, including 393 male participants, completed the trials, with 255 allocated to the intervention group and 253 assigned to the control group. In some studies, L-arginine has been used in cardioplegia, while in others, it has been used as a supplement alone or in combination with other substances. The timing of marker measurements varied from 15 min to 72 h following the disposal of the aortic clamp. In studies that used l-arginine supplements, the follow-up of patients varied between 30 days and 6 months. The dose of L-arginine in cardioplegia varied from 1 to 15 g, and in the studies that used the supplement, its dose was 6.4 g per day alone. Additionally, in another study, it was administered at a dose of 7 g in combination with 7 g glutamine and 1.5 g of beta-hydroxy-beta-methylbutyrate. The mean age of the participants ranged from 52.73 to 65.8 years. According to Cochrane scoring, three papers were classified as high quality [1, 19, 20] while the other four studies were of low quality [7, 10, 17, 18]. We conducted subgroup analyses to account for potential differences between acute (e.g., during cross-clamping or within the first 24–72 h) and non-acute settings (e.g., 30 days to 6 months). This was done to ensure that any variation in the timing of L-arginine administration did not influence our overall conclusions. Four studies were conducted in acute [7, 17–19] and three in non-acute settings [1, 10, 20].

**Table 1:** Characteristics of included studies

Authors, Country (year)	Number of patients (Intv/Ctrl)	Mean age (Intv/Ctrl)	Male (Intv/Ctrl)	Study design	Setting	Intervention type	Dosage of L-arginine	Time measure	Data presented	Result (WMD,95% CI)	Quality assessment
Colagrande et al., Italy (2006) ADDIN EN.CITE [7]	65 (33/32)	64.3 ± 8.7, 65.8 ± 8.7	90%,84.3%	RCT	Acute	7.5 g L-arginine in 500 mL of cardioplegic solution	7.5 gr	Follow-up (CPK-MB and Troponin T = 48 h) (IL-6 = 15 min) after aortic clamp removal	CPK-MB, Tn T, IL-6, TNF-α	CPK-MB = 5.20 (-4.99 ,15.39), Tn T = -0.30 (-0.46, -0.14), IL-6 = -30.80 (-44.51, -17.09), TNF-α = -38.30 (-67.76, -8.84)	Low
Lucotti et al., Italy (2009) ADDIN EN.CITE [10]	30 (16/14)	65 ± 10, 64 ± 11	93.75%,92.85%	RCT	Non-acute	L -arginine (6.4 g/d) for 6 months	6.4 gr	6 months	IL-6 TNF-α	IL-6 = -2.52 (-4.80, -0.24), TNF-α = 2.80 (-3.57 ,9.17)	Low
Carrier et al., Canada (1998) ADDIN EN.CITE [19]	50 (25/25)	62 ± 9, 62 ± 2	80%, 80%	RCT	Acute	1 g of L -arginine	1 gr	72 h after aortic clamp removal TnI, 48 h for CK-MB	CPK-MB	CPK-MB = 2 (-1.94 ,5.94)	High
Andrews et al., Australia (2012) ADDIN EN.CITE [17]	53 (27/26)	58.3±11.7, 60.8±9.5	81.48%,69.23%	RCT	Acute	15 g.L-1 of L-arginine	15 gr	24 h after aortic clamp removal	Tn I		Low
Carrier et al., Canada (2002) [18]	190 (94/96)	62 ± 15, 63 ± 13	79.78%, 84.37%	RCT	Acute	L -arginine– enriched solution (7.5 g/500 mL)	7.5 gr	48 h after aortic clamp removal	Tn T	Tn T = -0.11 (-0.21, -0.01)	Low
Norouzi et al., Iran (2022) ADDIN EN.CITE [20]	60 (30/30)	59.00 (29.50), 55.00 (14.50)	70%, 50%	RCT	Non-acute	Combination of 7 g L-arginine, 7 g l-glutamine, and 1.5 g daily HMB	7	30 days	CPK-MB Tn T	CPK-MB = -36.30 (-55.83, -16.77), Tn T = -2.22 (-2.95, -1.49)	High
Norouzi et al., Iran (2022) ADDIN EN.CITE [1]	60 (30/30)	52.73 ± 15.59, 53.53 ± 13.26	70%, 50%	RCT	Non-acute	Combination of 7 g L-arginine, 7 g l-glutamine, and 1.5 g daily HMB	7	30 days	IL-6 TNF-α	IL-6 = -3.22 (-6.90,0.46) TNF-α = -45.37 (-118.08,27.34)	High

Abbreviations: Intv, intervention; Ctrl, control; RCT, randomized clinical trial; CPK-MB, creatine kinase-MB; Tn T, troponin T; IL-6, interlukin-6; TNF-α, tumor necrosis factor-alpha; HMB, beta-hydroxy‐beta‐methylbutyrate

### Meta-analyses findings

#### L-arginine impact on cardiac biomarkers

**Fig. 3:**
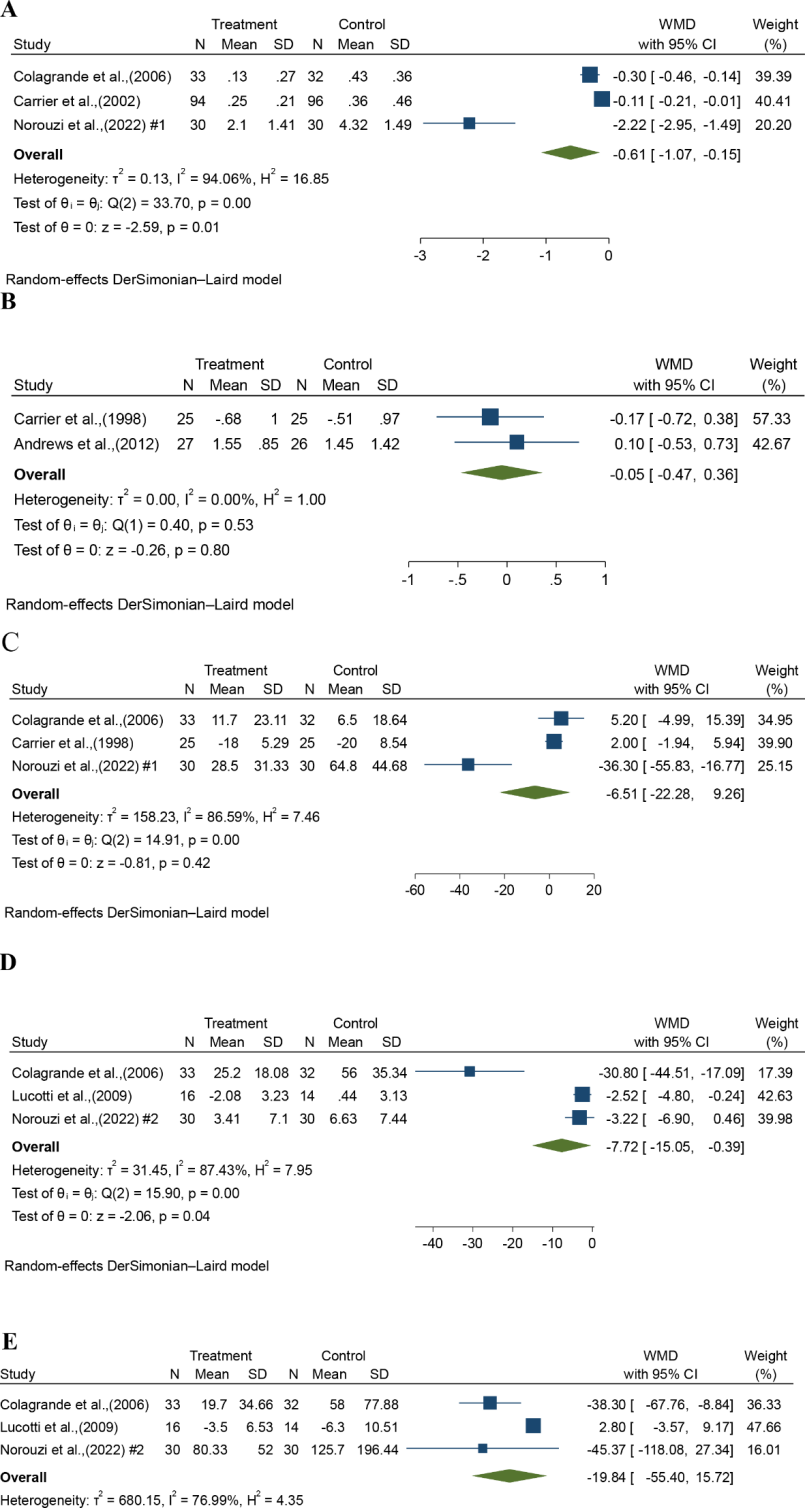
**A**– **E** The effect of L-arginine uses on (**A**) troponin T, (**B**) troponin I, (**C**) CPK-MB, (**D**) IL-6, and (**E**) TNF-α

A considerable influence of L-arginine in reducing troponin T levels (WMD = -0.61 ng/ml; 95% CI: -1.07, -0.15, Fig. 3A). We found significant heterogeneity between studies (*P* = 0.00, I^2^ = 94.06%). Regarding troponin I, a meta-analysis conducted on two RCTs revealed that L-arginine administration resulted in a decrease compared to the control group; however, these changes did not reach statistical significance (WMD= -0.05 µg/L, 95%CI: -0.47, 0.36, Fig. 3B). There was no heterogeneity among the studies (I^2^ = 0.00%, *P* = 0.53). In terms of CPK-MB changes, the meta-analysis of three RCTs indicated that L-arginine intake led to a reduction compared to the control groups, but no significant effect was seen (WMD = -6.51 IU/L, 95%CI: -22.28, 9.26, Fig. 3C). A considerable degree of heterogeneity was observed across studies (I^2^ = 86.59%, *P* = 0.00). Our subgroup analysis of acute and non-acute settings showed that the overall effects of L-arginine on the studied biomarkers were consistent across both groups. No significant differences were observed between the two settings, suggesting that L-arginine’s effect on cardiac and inflammatory biomarkers may be independent of the timing of administration in these patient populations (data are not shown).

#### L-arginine impact on inflammatory biomarkers

The effect of L-arginine on IL-6 was investigated using three effect sizes. Meta-analysis findings showed a considerable reduction in IL-6 levels due to L-arginine intake (WMD = -7.72 pg/ml; 95% CI: -15.05, -0.39, Fig. 3D). Considerable heterogeneity was observed (*P* = 0.00, I^2^ = 87.43%). Regarding TNF-α changes, the meta-analysis of three RCTs demonstrated a reduction in TNF-α levels with L-arginine intake in comparison with the control groups, but there was no statistically significant influence (WMD = -19.84 pg/ml, 95%CI: -55.40, 15.72, Fig. 3E). Significant heterogeneity was observed among the trials (I^2^ = 76.99%, *P* = 0.01).

### Sensitivity analysis

**Fig. 4:**
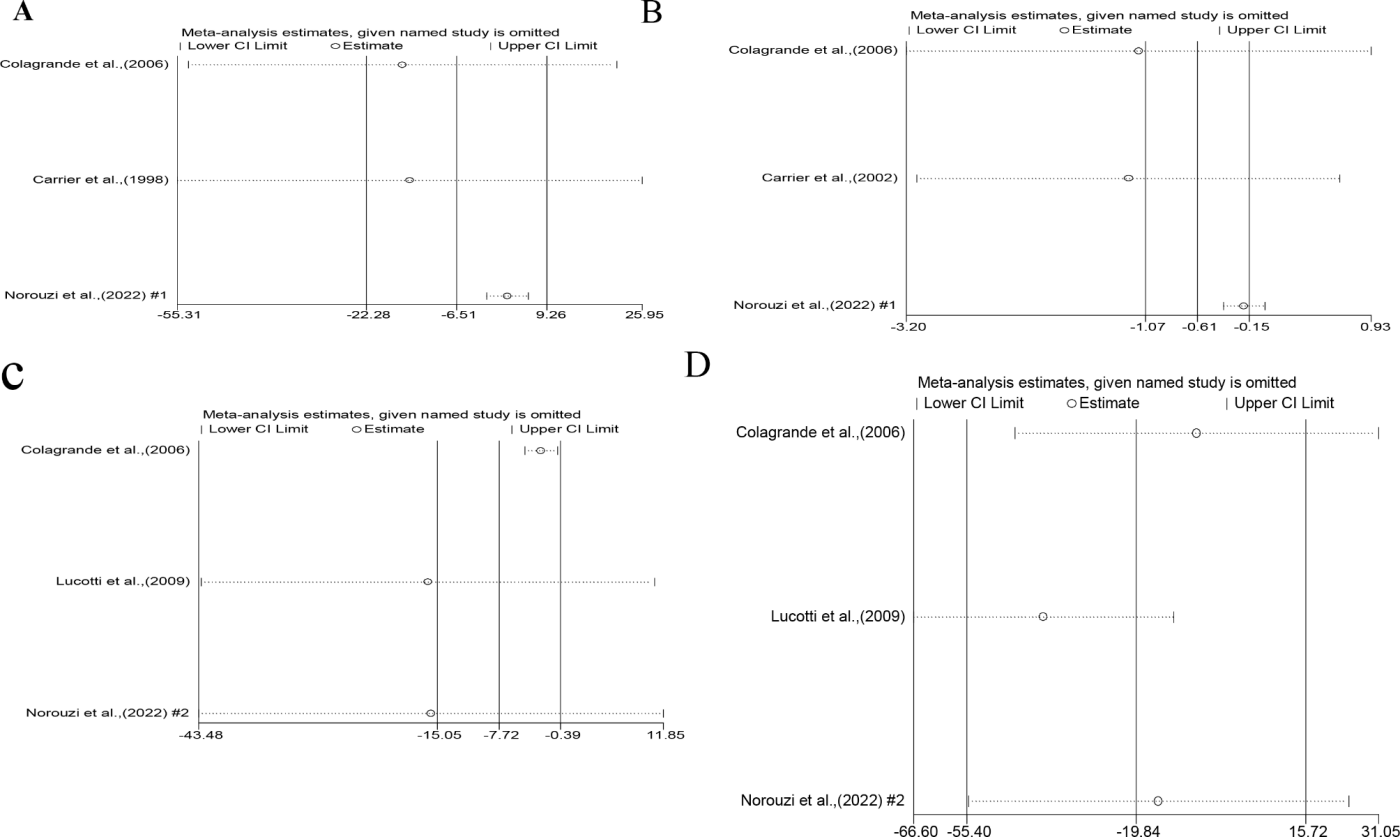
**A**-**D** The effect of one-by-one trial in the association between L-arginine use and cardiac and inflammatory markers using sensitivity analysis. (**A**) CPK-MB, (**B**) troponin T, (**C**) IL-6, and (**D**) TNF-α

Considering the identified heterogeneity among the studies, a sensitivity analysis was performed for each marker to account for this variability. Nevertheless, systematic exclusion of individual studies did not yield any noteworthy alterations in the overall combined WMD for CPK-MB (Fig. 4A). However, the sensitivity analysis revealed that the exclusion of articles by Colagrande et al. [7]. (WMD= -1.13, 95%CI: -3.19, 0.93) and Carrier et al. [18]. (WMD= -1.22, 95%CI: -3.10, 0.65) influenced the collective L-arginine effect on troponin T levels, rendering it statistically non-significant (*P* > 0.05) (Fig. 4B). Furthermore, the findings from the sensitivity analysis demonstrated noteworthy alterations in terms of the impact of l-arginine on inflammatory markers following the removal of certain RCTs. The exclusion of articles by Norouzi et al. [1]. (WMD= -15.81, 95%CI: -43.48, 11.84) and Lucotti et al. [10]. (WMD= -16.18, 95%CI: -43.16, 10.79) changed the overall effect of L-arginine on IL-6 levels to non-considerable (*P* > 0.05) (Fig. 4C). Additionally, sensitivity analysis indicated that the exclusion of the article by Lucotti et al. [10]. resulted in a considerable L-arginine impact on TNF-α concentration (WMD= -39.22, 95%CI: -66.60, -11.99) (Fig. 4D).

### Publication bias

We did not find any indications of publication bias across the articles included in our investigation of the impact of L-arginine on cardiac and inflammatory markers. This conclusion was reached after conducting Begg’s test (CPK-MB: *P* = 0.117, IL-6: *P* = -1.57, TNF-α: *P* = 0.602) and Egger’s tests (CPK-MB: *P* = 0.513, IL-6: *P* = 0.195, TNF-α: *P* = 0.272), except for the analysis of troponin T. In the case of troponin T, the results of the Egger’s test suggested the possibility of publication bias (*P* = 0.012). Consequently, a trim test was performed to address this issue, but it revealed no substantial alteration in the data.

## Discussion

This meta-analysis examined the effectiveness of L-arginine in decreasing myocardial stress and myocardial damage in subjects undergoing CABG surgery, focusing on the release of CK-MB, troponin I, troponin T, and myocardial cytokines. The pooled analysis showed that L-arginine caused a significant decline in troponin T and IL-6 levels. However, no significant effect was observed on TNF-α, CPK-MB, and troponin I.

The cardioprotective impacts of L-arginine have been well-documented in vivo [6]. A study by Carrier et al.. used L-arginine as an additive to cardioplegia during cardiac surgery for the first time [19]. In their study, the addition of 7.5 g of L-arginine to cardioplegia resulted in reduced levels of troponin T [18]. Previously, the same group tested a lower L-arginine dosage of 1 g, which was found to be safe but not efficacious [19]. Additionally, another study administered 15 g of L-arginine and found no impact on troponin I levels [17]. However, conflicting results have been observed when L-arginine is used as a myocardial protective substance. Animal studies have indicated that at concentrations of 4 mmol/l, L-arginine is effective and harmless when delivered in cardioplegia, but at concentrations > 5 mmol/l, it is less effective [21]. Furthermore, in a human study involving L-arginine cardioplegic arrest with 1 g of L-arginine delivered through induction cardioplegia (resulting in a concentration of 7–8 mmol/l), no measurable difference in cTnI level was observed [19]. Contrary to these unfavorable results, other researchers have documented noteworthy decreases in cardiac creatinine kinase and troponin-T levels when employing considerably higher concentrations of L-arginine cardioplegia [7, 18, 22]. Kronon et al.. conducted a study [23] to address the potential adverse effects of L-arginine overdose in cardioplegia. They’re in vitro investigation demonstrated that supplementation with L-arginine provides advantages when administered at a low dosage of 4 mmol, whereas harmful effects were observed at a high dose of 10 mmol/l. The researchers hypothesized that the enhanced production of peroxynitrite, an oxygen free radical generated from NO, might be responsible for the heightened oxidative damage associated with a higher dose of L-arginine. Some trials have examined the influence of oral administration of L-arginine on postoperative inflammation and cardiac factors [1, 20]. These trials concluded that L-arginine exerts a cardioprotective effect by reducing inflammatory and cardiac factors. One possible mechanism is the effect of arginine deficiency on blood cell maturation. Research suggests that arginine deficiency can impair mitogenic reactions and cell-mediated immune responses in peripheral T cells [24].

L-arginine, precursor of NO, acts as an NO donor and plays a crucial role in the regulation of vascular function [25]. The release of NO during the administration of L-arginine throughout cardioplegia is linked to reduced endothelial activation and improved myocardial preservation during arrest and reperfusion [7]. The protective effects of L-arginine are also associated with its ability to reduce inflammatory responses, limit endothelial injury from oxygen radical production, and decrease the generation of free radicals originating from oxygen [25–27].

While previous studies have suggested that the effects of L-arginine may vary between acute and non-acute settings, our subgroup analysis revealed no significant differences in the overall effects of L-arginine. However, we acknowledge that further studies with larger sample sizes focused on each setting individually are warranted to confirm this finding. Notably, in light of previous studies, such as the RCT that examined L-arginine in post-infarction patients [28], where treatment was associated with early deaths and no improvement in vascular stiffness or ejection fraction, caution is warranted when considering L-arginine therapy in any post-surgical setting, including CABG patients. While the pathophysiology of post-myocardial infarction may differ from post-CABG recovery, the potential risks associated with L-arginine, particularly in the months following surgery, should be carefully evaluated before routine use in clinical practice.

The “no-reflow” phenomenon refers to a condition where, despite restoring blood flow to a previously ischemic area, there is inadequate perfusion of the tissue. This occurrence is marked by impaired functioning of the endothelium, deficiency in NO, and liberation of free radicals originating from oxygen. L-arginine supplementation can help alleviate the no-reflow phenomenon by increasing NO synthesis and liberation, leading to decreased coronary vascular resistance and improved blood flow [7]. By improving endothelial function, reducing platelet activation, and neutralizing superoxide anions, L-arginine helps prevent oxidative myocyte necrosis and enhances overall tissue perfusion during reperfusion by improving endothelial function, reducing platelet activation, and neutralizing superoxide anions. These mechanisms collectively contribute to the favorable impact of L-arginine on cardiovascular factors in individuals undergoing CABG surgery. Therefore, L-arginine supplementation, either orally or in cardioplegia, might be recommended as an effective approach to mitigate postoperative complications in cardiac patients undergoing CABG.

Our study represents the first systematic review and meta-analysis to assess the impact of l-arginine on inflammatory and cardiac factors in patients undergoing CABG. However, this study has limitations that need to be considered. The primary limitation of this study was the high heterogeneity that was observed among the included studies. However, we conducted sensitivity analysis to strengthen the reliability and validity of our findings. Moreover, one of the limitations of our study is the heterogeneity of the data, which may contribute to the inconsistent findings between Troponin T and Troponin I. While Troponin T levels were significantly reduced, there was no significant change in Troponin I. The biological reason for this discrepancy is unclear and may be related to variations in study design or patient characteristics. Further studies with more uniform protocols and larger sample sizes are necessary to validate these findings. Another limitation is the limited number of RCTs on the subject, which makes it difficult to identify the source of heterogeneity according to some moderator variables. Moreover, a major drawback of the studies reviewed is that they all employed approximately the same dose of L-arginine, leaving it unclear what the effective dose might be. To determine the optimal dose or whether the supplement is ineffective, dose-response studies are required. Last but not least, a significant limitation of our study is the lack of long-term safety data for L-arginine use in the months following CABG surgery. Previous studies, such as the RCT in post-infarction patients [28], have raised concerns about increased mortality with L-arginine use in such populations. Although our study focuses on a different clinical setting, these findings suggest the need for further research to ensure patient safety before L-arginine can be recommended post-CABG. Therefore, caution should be exercised when interpreting the results of this meta-analysis.

## Conclusion

The pooled estimates from our systematic review and meta-analysis indicate that L-arginine can reduce myocardial stress by decreasing the release of local cytokines. Additionally, our findings demonstrate the protective effects of l-arginine, as evidenced by a considerable decline in the release of troponin T in individuals undergoing CABG surgery.

## Data Availability

The data that support the findings of this study are available from the corresponding author upon reasonable request.
